# The management of primitive retroperitoneal tumors - problems of clinical, imagistic diagnosis, and treatment

**Published:** 2008-08-15

**Authors:** Ovidiu Bratu, Victor Madan, Florin Rusu, Cristian Ilie, Dan Mischianu, Rodica Barla, Petre Hoara, Silviu Constantinoiu

**Affiliations:** * Urology Clinic – Central Military Emergency Universitary Hospital “Dr. Carol Davila” Bucharest; ** Surgery Clinic – Hospital “Sf. Maria” Bucharest

## Abstract

Retroperitoneal tumors, whether primary or resulting from the metastasis of other tumors, are a real challenge for the surgeon, as regards their diagnosis and treatment.

They are relatively rare, under 0.2 % of the total number of tumors.

The clinical examination of retroperitoneal tumors is uncharacteristic and misleading, consisting mainly in palpation of the tumor proper and in pain. The other signs and symptoms often result from the affected neighboring organs.

The imagistic investigations used in diagnosing retroperitoneal tumors are ecography, intravenous pielography, computed tomography, MRI, PET/CT.

The main treatment is the surgical one, consisting either in the total or partial excision of the tumor, or in biopsic material samples with a view to making a histopathologic diagnosis.

Post-operatory evolution depends mainly on the thoroughness of the surgical treatment, that is the complete excision of the tumor, which increases the chances of survival, while lowering the risk of relapse.

Primitive retroperitoneal tumors (PRPT) are of interest for several surgical branches in point of diagnosis and therapy, falling to an equal extent into the scope of the general surgeon, the urologist and the gynecologist.

Whether discovered *de novo* or resulting from a metastatic primary tumor in another organ, their diagnosis and treatment raise particular problems for the clinician.

The article below will dwell specifically on these problems.

Ever since the term of retroperitoneal tumor was introduced by Lobstein in 1842 [**2**] and up to the first exereses, the treatment has not undergone significant changes. Despite technological progress, immunology and genetics, surgery has remained the last recourse in diagnosing, treating and considerably extending the patient’s life.

The retroperitoneal area, metaphorically labeled by surgeons “no man’s and, at the same time, everybody’s land”, requires vast surgical knowledge - intestinal and urinary tract reconstruction surgery, as well as partial blood vessel resection surgery [**1**,**4**].

We will insist neither on the anatomy of this area, nor on already known clinical and histological aspects, but we will briefly mention them below.

Primitive retroperitoneal tumors represent a relatively rare oncological pathology, under 0.2% of the total number of tumors[**1**,**3**]. They appear mostly in patients between 40 and 70 years old, but they may also be found at extreme ages (babies or elderly persons), without any difference between sexes.

Given the profusion of lax tissue and the specific anatomic elements of the retroperitoneum, there are numerous histological types of retroperitoneal tumors. The highest number of cases appear to be lymphomas and sarcomas in adults, and neuroblastomas in children [**1**,**2**].

## Clinical Signs

The clinical signs of these tumors are totally uncharacteristic and misleading. Apart from the presence of a large tumoral mass, accessible to lombo-abdominal palpation, the signs and symptoms are frequently the result of affected neighbouring organs. Interpreted by a specialist lacking information and/or specific knowledge, these signs and symptoms may easily lead to a wrong diagnosis (See **Table 1**).

**Table 1:** Signs and symptoms encountered in PRPT

Signs and symptoms	Clinical manifestation
Palpable tumoral mass	- Mostly hard, adheres to the neighbouring tissue, modifies the symmetry of the abdomen
Pain	- Vague, diffuse, often located on sides
Urinary signs	- Renal colic, macroscopic hematuria, disuria, polakiuria, vesical spasms
Digestive signs	-Postprandial plenitude, dyspeptic syndrome, epigastralgy, vomiting, constipation, diahorrea etc
	-Sub-oclusion or occlusion syndromes, hematemesis and/or melena
	-Icteric syndrome
	-Portal or splenic stasis syndrome with portal hypertension, splenomegalia, esophageal varices, ascitis
Neurological signs	-Metrical and sensitivity disturbances (paraplegy, areflexy, hypoestesy, sphincter incontinence by Cauda Equina syndrome etc
Vascular signs	-Oedema and varices in the lower limbs and the genital organs (varicocele, vulvocele (1,6), with the development of cavo-cave collateral circulation
Fever syndrome	-Occurs mostly in cases of intra-tumoral necrosis
Hormonal secretion	-Hypoglycemiant clinical forms of retroperitoneal tumors, with secretion of “insuline-like” substances (mesodermic tumors)
	- clinical forms with arterial hypertension (catecholaminic secretion, for instance neuroblastomas) or with cortyzol or aldosteron secretion (sarcomas).

## Imagistic investigations

Nowadays indispensable, the imagistic investigations used in diagnosing retroperitoneal tumors are: ecography, intravenous pielography, computed tomography, MRI, PET/CT.

Abdominal ecography may or may not provide information as to the existence of a retroperitoneal tumoral formation. Its advantage is that it is available in any modern medical facility, it is easy to perform, radiation-free, it may be repeated and it may lead the physician to a diagnosis, at last in the initial stage.

Intravenous pielography is an extremely useful investigation in the case of large retroperitoneal tumors which compress or affect the urinary ducts and/or the kidney. It may either reveal hydronephrosis by tumoral extrinsic compression of the urinary tract or it may expose an urographically mute kidney by tumoral invasion of the renal pedicle.

In the current stage of knowledge, the CT, MRI and PET/CT investigations of the retroperitoneal area are decisive. They are able to disclose the size, content, relations and invasion of the tumor. In certain cases, they may also estimate the histological type of the tumor (angiomyolipoma) or they may indicate the possibility or impossibility of radical tumor exeresis. Nevertheless, sometimes they may also be misleading (depending on the experience of the imagistic specialist). [**4**]

The PET/CT investigation is very useful in detecting and treating tumor relapses after intended radical surgery and may provide surgical treatment even before the tumor becomes clinically manifest.

## Laboratory examination

As regards current laboratory tests, they are altogether uncharacteristic, devoid of any specific element. The biochemical constants may be altered in the case of tumor invasion in another organ. For instance, the bilirubin value will rise, followed by icterus, in case of PRPT with invasion of the biliar tract or uremia (seric creatinine↑, seric urea↑) in the case of an affected kidney. [**1**].

Immunologically speaking, in the current stage of knowledge there is no specific tumoral marker. In certain histo-pathological types an increasing quantity of antigens (CA 19-9, carcino-embryonary antigen, alpha-fetoprotein etc) or other interleukine-type substances have been revealed, as well as a decreasing number of NK lymphocytes (CD16+CD56+) (natural killer) or variations of the number of T helper lymphocytes (CD3+CD4+) or T suppressors/cytotoxic(CD3+CD8+), without any obvious cause-effect connection between the retroperitoneal tumor and the emergence of these markers or lymphocytes. Yet, it is certain that, in most cases we have studied, there was a considerable immunological depression, as well as a decreasing number of T lymphocytes. Genetical studies have not revealed characteristic alterations either, the same oncogens present in other types of tumors being noticed in most cases.[**7**]

Some of the signataries of this article would very much like to point out, with the support of specialists in genetics and medical immunology, the existence of some tumor-predictive markers or to determine the presence/absence of protooncogens capable of causing the development of a tumor or of preventing its evolution.

## Treatment

The treatment of retroperitoneal tumors is mostly surgical.

The most frequent surgical approach is front-abdominal, transperitoneal, large enough to allow an easy access to the tumor, possibly to allow other interventions that could not be suspected before surgery (partial resection of neighbouring organs, intestinal anastomosis, nephrectomy etc)[**1**,**2**,**3**,**4**]

The surgical intervention aims at a complete excision of the tumor, within oncologically safe limits. If this is not possible, then the surgeon will resort to tumoral cytoreduction, if the local situation allows it. If neither of the variants above is possible, because an exeresis would be impossible or because there would be high risk of intraoperatory bleeding, the surgeon will perform tumoral biopsy, in order to determine the cytopathological type of the tumor and will refer the case to the oncologist.

Radiotherapy and chemotherapy are adjuvant treatments with modest results in the case of primitive retroperitoneal tumors.

Radiotherapy may be performed pre-surgery (when the tumor is initially considered inextirpable and the treatment aims at reducing the size of the tumor, with a view to further surgery) or post-surgery (in case of incomplete exereses, with remaining tumoral tissue in the area).[**1**,**2**]

In the cases we have been faced with, we have witnessed a spectacular result in the situation of a gigantic extragonadal seminoma (histopathological result obtained by intraoperatory tumoral punction), initially inextirpable but almost 10 times smaller after radiotherapy, which later on made possible its total exeresis.

Chemotherapy has even lesser results than radiotherapy. For all the complexity of the existing therapeutic patterns, it does not provide significant long-term survival, while highly toxic and with numberless secondary effects. [**2**]

In the following pages we will provide clinical and imagistic examples of some cases of retroperitoneal tumors.

**Case 1**

Patient B.S., female, 57, was hospitalized with left-side lombar colics and diffuse permanent pain in the left pelvic member. Being diagnosed with fibromatose uterus, in 2004 she had undergone hysterectomy with bilateral anexectomy (histopathological result: uterine fibrome). Following paraclinical investigations, the diagnosis was that of invading retroperitoneal tumor in vertebrae L4,L5, compressing the left ureter. We placed a double J ureter stent on the left side, to decompress the left kidney. We performed tomographically guided biopsic punction. **The result: poorly differentiated retroperitoneal fibrosarcoma.** The neurosurgical examination established that the case was inoperable (a possible radicular decompression in the pelvic member), so the pacient was referred to an oncologist.

**Fig.1,2:**
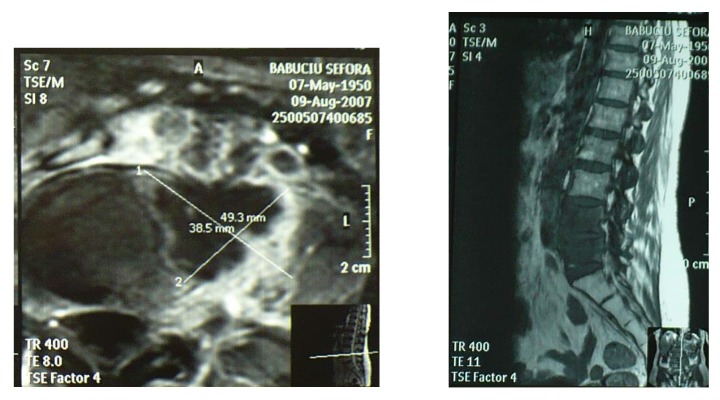
MRI images – invading retroperitoneal tumoral formation in vertebrae L4,L5

**Fig.3:**
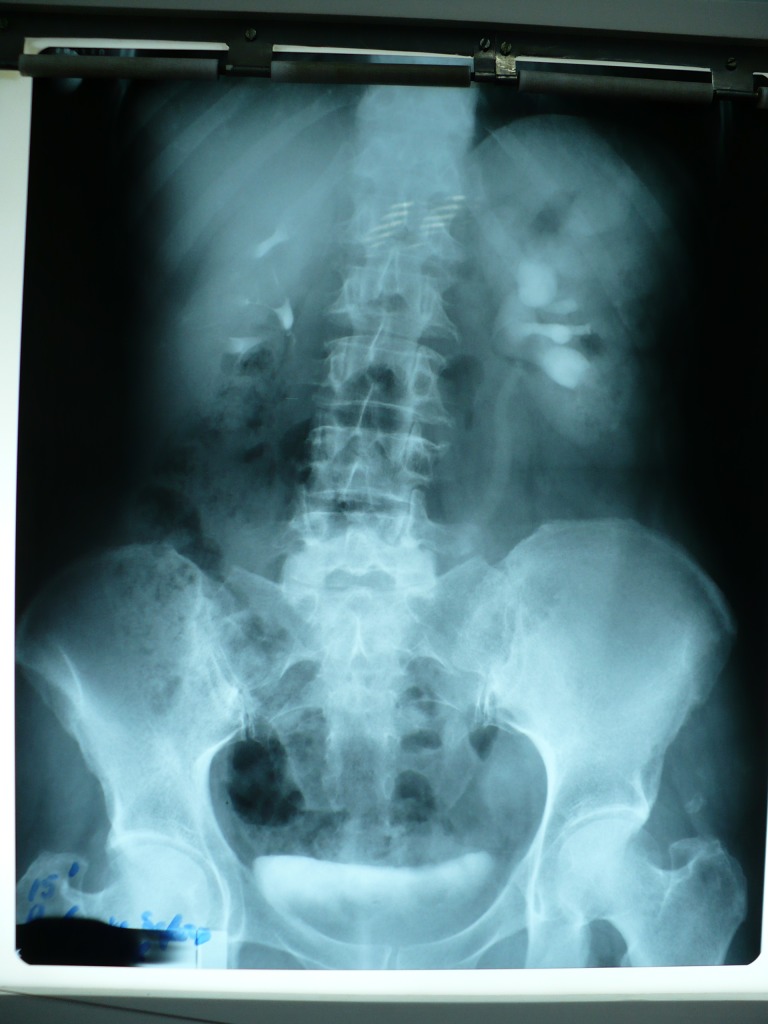
L.V.P. Left ureterohydronephrosis, II-III degree, caused by tumoral compression on the lower lombar ureter.

**Case 2**

Patient M.M., female, 72, was hospitalized for diffuse pain in the right side. The tests (leucocyte formula, blood biochemistry) were within normal limits. The abdominal CT investigation revealed a 6/4 retroperitoneal formation, well-delimited in relation to the lower cave vein. Surgery was performed by frontal approach, with exeresis of the tumoral formation. **Histopathological result: well-differentiated carcinoma**, without any possibility of determining the starting point, not even immunohistochemically. The CT investigation, 6 months after surgery, did not reveal tumoral relapse.

**Fig.4:**
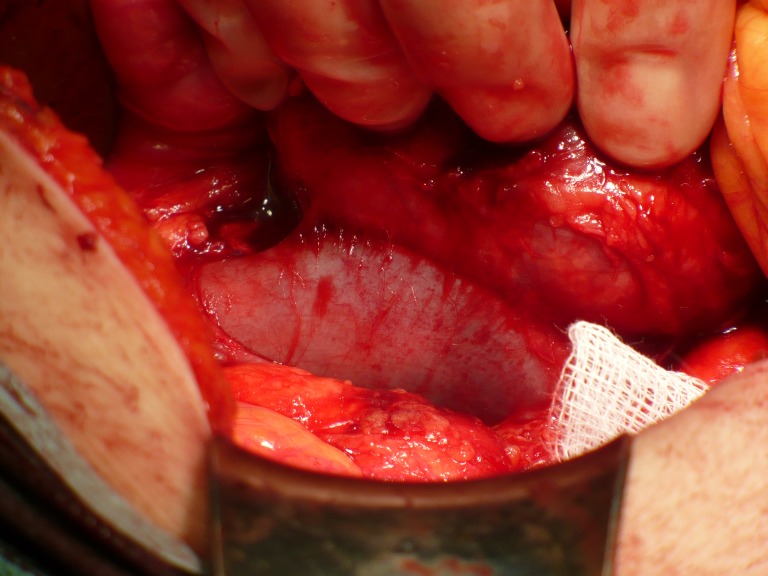
The retroperitoneal tumor in relation to the lower cave vein

**Fig.5,6:**
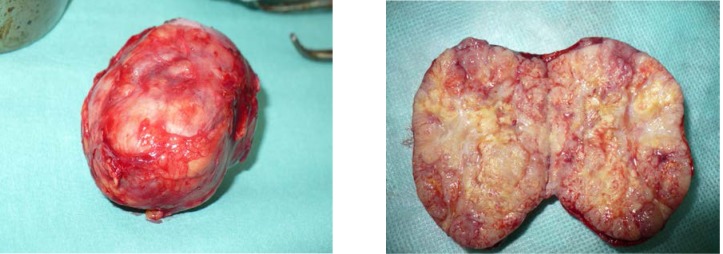
Post-surgery images of the excised piece

**Case 3**

Patient H.M., female, 52, operated on 2 months before, outside the capital, for a supposed right-side ovar cyst, was hospitalized with abdominal pain in the right pelvic area, deep trombophlebitis in the right pelvic member, subfebrility and moderate loss of weight (7 kg in 4 months). The paraclinic investigation revealed leukocytosis (20 000 leukocytes/mm3). The ecography showed right ureterohydronephrosis, IV degree. The MRI investigation (the patient was allergic to iodine, so that intravenous pielography and CT investigation were out of question) pointed out a large formation, 13/10cm, located in the pelvis, infiltrating the iliac bone and muscle, compressing the right external iliac vascular axis and including the right ureter. Surgery was performed by frontal approach and median incision, sub- and supraombilical, revealing a large formation, apparently developed in the right ovar, with a 3/3 cm tumor necrosis area on the frontal surface. The right ureter was incorporated into this formation and practically strangulated. The formation was extremely adherent to the neighbouring tissues, without a dissection plane. About 100 ml yellowish liquid was extracted, out of which some cytologic samples (examination under way) and a double J stent was installed intrasurgically. Post-surgery, thrombophlebitis came under control by steady anti-coagulant treatment. **Histopathological result: anaplasic pelvisubperitoneal fibrosarcoma** with central tumor necrosis. The patient was referred to the oncologist.

**Fig.7:**
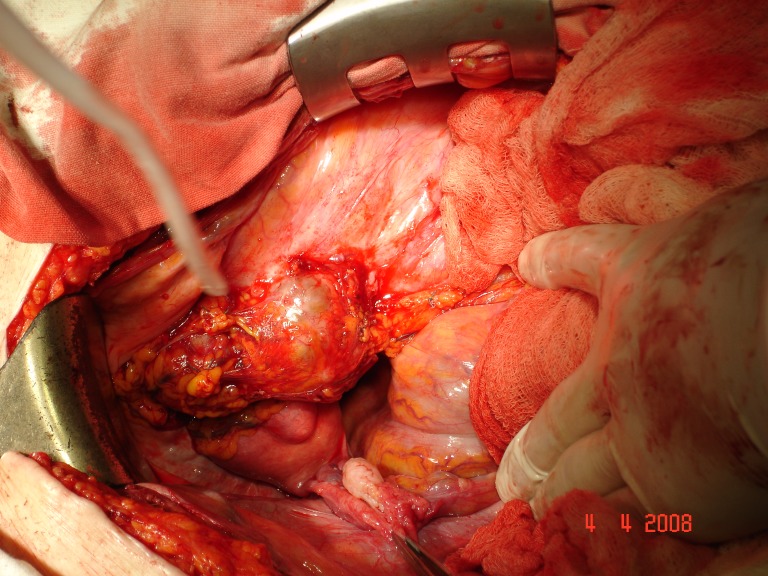
The retroperitoneal (pelvisubperitoneal) tumor and its relation to the pelvic organs

**Fig.8:**
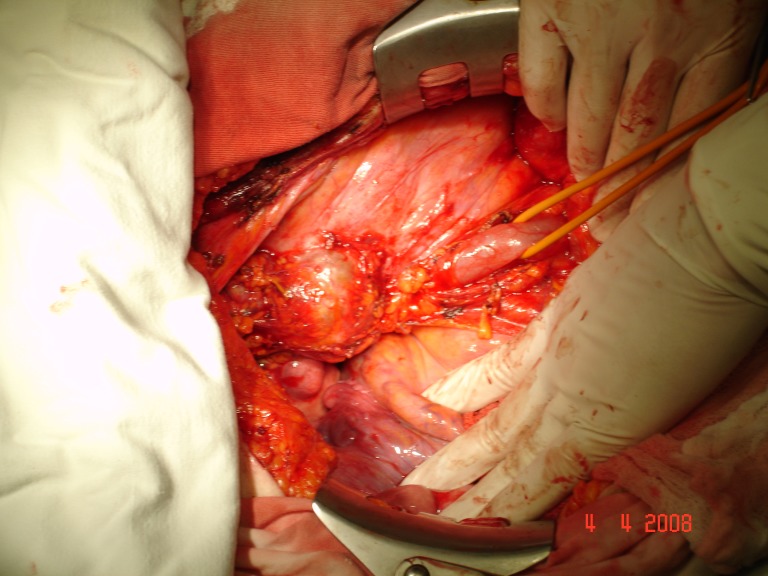
Isolating the pelvic ureter from the retroperitoneal tumoral mass

**Fig.9:**
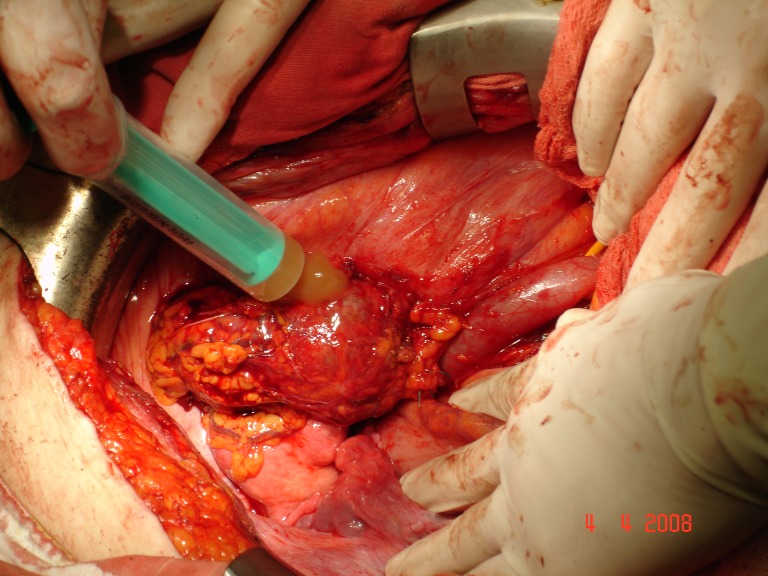
Evacuating the tumoral content, with further partial exeresis of the tumor

**Fig.10:**
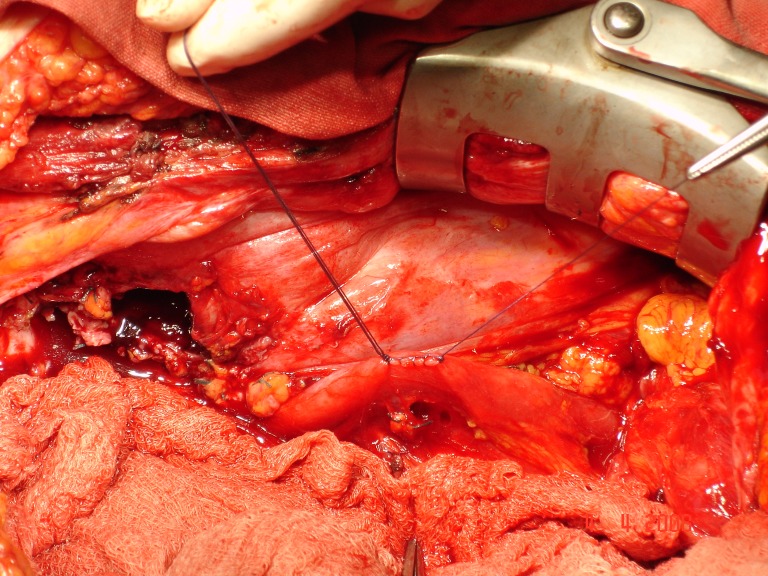
Suturing the ureter after intrasurgical installation of a double J ureteral stent

**Case 4**

Patient M.G., male, 75, was hospitalized for enlarged abdomen and intestinal transit disorder. Bioumoral parameters within the normal limits. The CT investigation revealed a large tumoral formation in the abdomen, extending to the pelvic area and compressing the vesical cap. Intrasurgically, the formation was found to be adherent to the intestinal anses, with a cleavage plane, so that the tumor could be completely excised, after being evacuated (about 5 kg yellowish jelly-like liquid). **Histopathological result: retroperitoneal pseudomyxom**.

**Fig.11,12:**
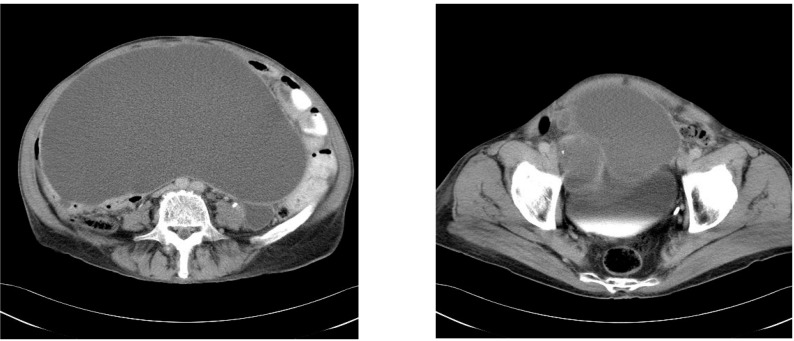
CT images –Large abdominal tumoral formation extending to the vesical cap

**Fig.13:**
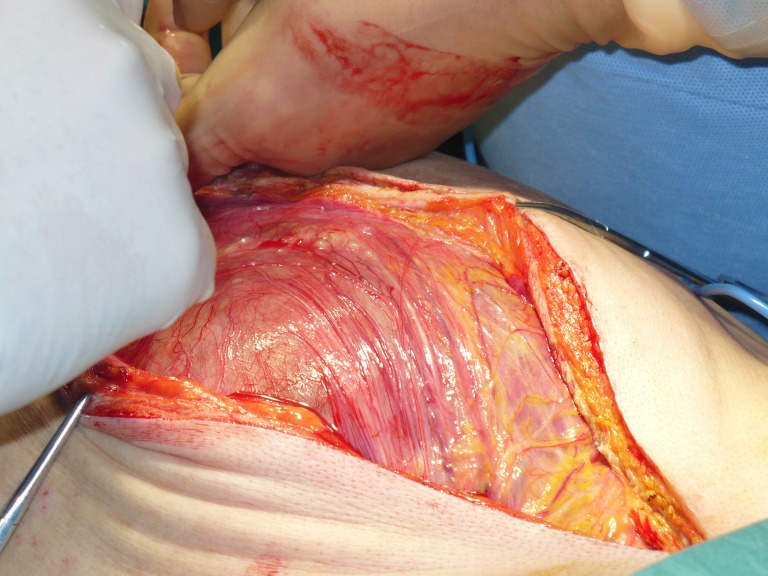
Laparotomy revealing the tumoral formation

**Fig.14:**
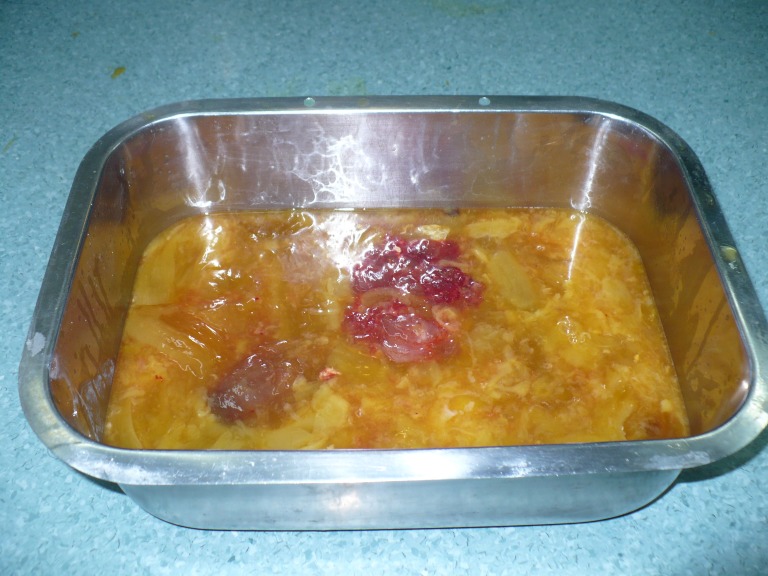
Tumoral content (yellowish jelly-like liquid)

**Fig.15:**
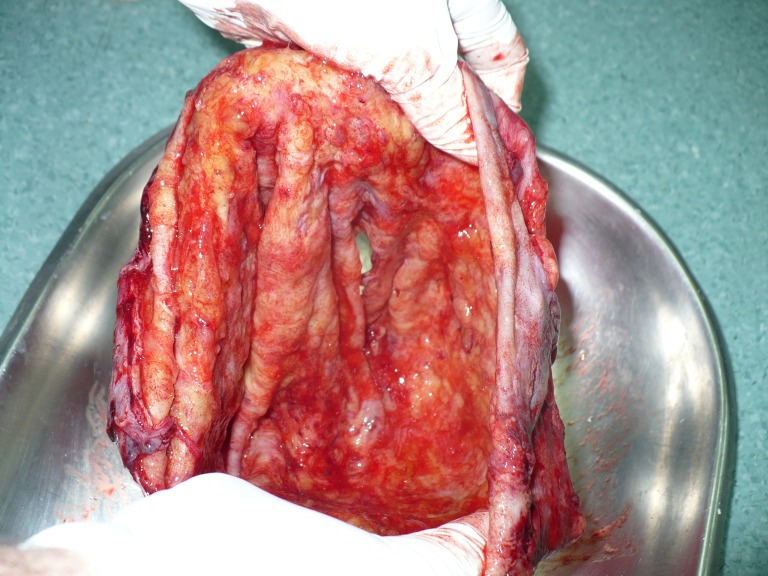
The tumor capsule

**Case 5**

Patient M.F., female, 38, with multiple surgical antecedents, was hospitalized with nonfunctional left kidney, discovered by ambulatory intravenous urography. Following a punction performed into a mediastinal tumoral formation, she was diagnosed with Castleman’s disease and followed immunosuppresive treatment for 3 years (cyclophosphamide). The abdominal CT investigation revealed a 7/7 cm retroperitoneal tumoral formation. Intrasurgically, the retroperitoneal tumoral formation was discovered between the lombar spine and the big blood vessels, very adherent to the latter, with no possibility of a radical intervention. The left ureter pervaded into this hard, fibrous tumoral mass. About 4 cm of the lombar ureter englobed into the tumor was excised, then its upper and lower ends were joined by termino-terminal anastomosis and a ureteral stent was installed intrasurgically. Biopsic samples were prelevated from the tumor. **Histopathological result: retroperitoneal fibrosis **(Ormond’s disease). The control I.V.P. undertaken 30 days after surgery showed bilateral renal functionality.

**Fig.16:**
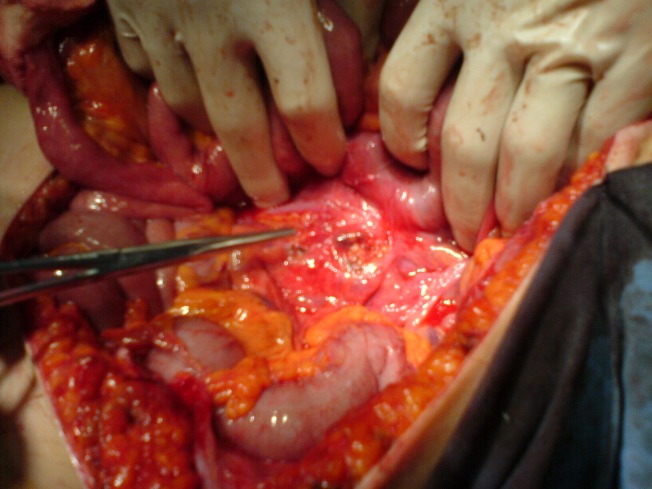
Intrasurgery image of the retroperitoneal tumoral formation located between the lombar spine and the big blood vessels, and incorporating the lombar ureter.

**Fig.17,18:**
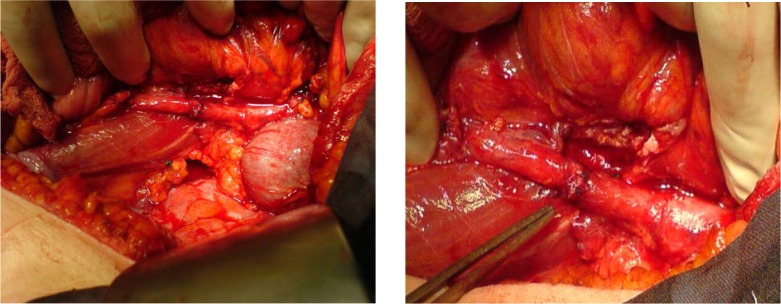
Intrasurgery images – termino-terminal anastomosis of the ureter extremities, after excision of the intra-tumoral ureter and intrasurgery installation of a double J ureteral stent

## Comments

The postsurgery evolution of these tumors depends mainly on the thoroughness of the initial surgical intervention. If the tumoral mass is wholly excised, this being performed within oncologically safe limits, then the risk of relapse is low and the survival rate will increase [**1**,**2**,**4**]. In the cases we were face with, this was possible in very few situations. More often than not we were able to only prelevate biopsic samples from the tumor tissue, in order to determine its histopathological type and a further oncological approach.

In our estimation, retroperitoneal tumors are a special branch of oncologic surgery. Being located deep in the retroperitoneum and frequently invading the neighbouring organs and the big blood vessels - so that not only tumor exeresis is mandatory but often also enterectomies, splenectomies, nephrectomies, colectomies, partial cave vein resections etc - this pathology is a genuine challenge for the surgeon[**3**,**4**].

The hope for an early diagnosis and – why not? – for treatment lies now with geneticians.

Retroperitoneal tumors are for urologic, gynecologic and abdominal surgeons (our deep conviction is that a surgeon has to approach all these areas) a permanent challenge, and the notion of “oncology surgeon” is meaningless in respect to the three specialized domains mentioned above.

For all these specialists, but mostly for the urologic surgeon, retroperitoneal tumors are no longer “terra incognita”!
